# Correlation of immune makers with HPV 16 infections and the prognosis in oropharyngeal squamous cell carcinoma

**DOI:** 10.1007/s00784-023-04926-2

**Published:** 2023-03-08

**Authors:** Yingying Zhu, Xiaoli Zhu, Wenwen Diao, Zhiyong Liang, Zhiqiang Gao, Xingming Chen

**Affiliations:** 1 Department of Otolaryngology-Head and Neck Surgery, Peking Union Medical College and Chinese Academy of Medical Sciences, Peking Union Medical College Hospital, No.1, Shuaifuyuan, Beijing, 100730 Wangfujing China; 2 Department of Pathology, Peking Union Medical College and Chinese Academy of Medical Sciences, Peking Union Medical College Hospital, Beijing, 100730 China

**Keywords:** Head and neck cancer, Oropharyngeal squamous cell carcinoma, Human papillomavirus, Programmed death ligand-1, Prognosis

## Abstract

**Objectives:**

This study aims to investigate the association of immune markers with high risk human papillomavirus 16 (HPV 16) infection status and to evaluate the prognostic value of programmed death ligand-1 (PD-L1) in patients with oropharyngeal squamous cell carcinoma (OPSCC).

**Materials and methods:**

This retrospective study collected 50 cases of HPV positive and HPV negative OPSCC from January 2011 to December 2015. The correlation of CD8 + tumor infiltrating lymphocytes (TILs), programmed death-1 (PD-1), and PD-L1 expression with HPV 16 infection status was analyzed via immunofluorescent staining and quantitative real-time PCR.

**Results:**

There was no significant difference in the baseline data between the two groups. Patients with HPV + OPSCC had better prognosis compared to HPV − patients (5-year overall survival [OS], 66% vs. 40%, *P* = 0.003; 5-year disease specific survival [DSS], 73% vs. 44%, *P* = 0.001). The expressions of immunity related makers were significantly higher in the HPV + group than the HPV − group (CD8 + TIL: *P* = 0.039; PD-L1: *P* = 0.005; PD-1: *P* = 0.044). Positive CD8 + TIL and PD-L1 were independent factors for better prognosis of OPSCC (DSS, *P* < 0.001; OS, *P* < 0.001, respectively). Kaplan–Meier survival analysis indicated that patients with TILs of high HPV + /CD8 + expression were more likely to have better prognosis than those with TILs of low HPV + /CD8 + expression (DSS, *P* < 0.001; OS, *P* < 0.001), TILs of high expression of HPV − /CD8 + (DSS, *P* = 0.010; OS, *P* = 0.032), and TILs of low expression of HPV − /CD8 + (DSS, *P* < 0.001; OS, *P* < 0.001). Furthermore, HPV + /PD-L1 + OPSCC patients had significant better prognosis compared to patients with HPV + /PD-L1 − (DSS, *P* < 0.001; OS, *P* = 0.004), HPV − /PD-L1 + (DSS, *P* = 0.010; OS, *P* = 0.048) and HPV − /PD-L1 − (DSS, *P* < 0.001; OS, *P* < 0.001).

**Conclusions:**

HPV + OPSCC had a significantly better prognosis, and PD-L1 expression was elevated in HPV + OPSCC. PD-L1 positivity might be related to the better prognosis of HPV + OPSCC.

**Clinical relevance:**

This study provides a theoretical basis and baseline data for the application of immune checkpoint inhibitors in head and neck tumors.

## Introduction

Oropharyngeal carcinoma is one of the most common malignant tumors in head and neck, and more than 90% of them are squamous cell carcinoma [1]. Human papillomavirus (HPV) has been recognized as an important infectious factor for oropharyngeal squamous cell carcinoma (OPSCC) [2]. Globally, about 30.4% of OPSCCs (which mainly comprises the tonsils and base of tongue sites) are caused by HPV infection each year [3]. Relevant studies have found that the clinical prognosis of patients with HPV + OPSCC is better than those who are HPV − among western people, especially HPV 16, a hypotype of HPV [4–7]*.* Besides, the response to immunotherapy are different between patients with HPV + and HPV − OPSCC [8, 9].

Tumor infiltrating lymphocytes (TILs) contribute to immune response in tumor immune microenvironment, and the infiltration degree of CD8 + TILs is correlated with the clinical prognosis in OPSCC [10]. In addition, programmed death-1 (PD-1) and its ligand, programmed death ligand-1 (PD-L1), are negative immunoregulatory factors discovered in recent years [11]. In head and neck cancer, multiple gene expression subtypes have been described, which also correlate with HPV, immune response, and possibly tobacco smoking [12]. High expression of PD-L1 in intra-tumoral immune cells and abundant CD8 + TILs in HPV OPSCC can identify subgroups of patients with excellent outcomes and provide additional prognostic value beyond existing staging systems [13, 14]. However, whether immune response is associated with survival of OPSCC with different HPV status is still not clear.

The objective of this study was to investigate the expression of CD8 + TIL, PD-1, and PD-L1 in OPSCC, and analyze their correlations with clinical prognosis of different HPV status.

## Materials and methods

This study retrospectively involved 100 cases of patients with OPSCC, which included 50 cases of HPV + OPSCC and 50 cases of HPV- OPSCC. The patients were recruited from Peking Union Medical College Hospital from 2014 to 2018, and they had been followed up for more than 5 years until death. The correlation of PD-L1 expression with HPV 16 infection status was analyzed using propensity score matching (PSM). The clinical parameter of patients was collected, including gender, age, living habit, and clinical stage. The pathological characteristics were also collected. All patients were graded according to the TNM grading criteria specified by the American Joint Committee on Cancer (AJCC) in 2010 [15]. All patients and their families have signed the informed consents. The Peking Union Medical College Hospital (PUMCH) Ethics Committee approved this study protocol (number: S-K795).

### Inclusion and exclusion criterion

Inclusion criteria: (1) all patients met the diagnostic criteria in the guidelines of The 2017 WHO Classification of Head and Neck Tumours [16]; (2) patients who did not receive immunotherapy; (3) patients who were diagnosed as OPSCC from the tonsils and base of tongue sites.

Exclusion criteria: Patients with the following diseases or conditions were excluded: (1) severe diseases such as immune system disease; (2) inability to cooperate; (3) other kinds of malignant disease; (4) infection in oropharyngeal or other inflammatory diseases that may affect the results of the study.

### HPV 16 DNA testing

The DNA in the tumor tissue samples were extracted using QIAamp DNA FFPE Tissue Kit (Qiagen Ltd, Hilden, Germany). Rotor Gene 6000 and SYBR Premix Ex Taq Kit (Perfect Real Time, Takara Bio, DRR063a, Japan) were applied for quantitative real-time PCR (qRT-PCR) for detection of HPV 16 DNA. Primers and FAM-MGB labeled probes (TaqMan) were designed and optimized to amplify the HPV16E6 region. The HPV 16-positive cervical cancer cell lines CaSki and SiHa (ATCC-LGC-HTB-35) were used as positive controls and calibrators for the assay. The detection threshold for HPV-positive status was set, and samples were deemed positive if the threshold was met in duplicate runs.

### CD8, PD-1, PD-L1, and PD-1/CD8 expression by immunofluorescent staining

Paraffin sections about 5 μm thickness were prepared by conventional methods, and then, immunofluorescence staining was performed after dewaxing. Antigen repair was performed in EDTA buffer in microwave. The slices were added primary antibody CD8 (dilution 1:1000), PD-1 (dilution 1:1000), PD-L1 (dilution 1:100), and incubated overnight at 4 °C. After sections were washed, fluorescent-labeled secondary antibody (dilution 1:300) was incubated, and then, FITC reagent (CD8 sections) and CY3 (PD-1 and PD-L1 sections) were dropped, and autofluorescence quencher was added after incubation in the dark. Finally, DAPI was re-stained. All the sections were sealed by fluorescence decay–resistant medium and recorded in fluorescent microscope, and each was viewed by two experienced pathologists who was blinded to the patients’ clinicopathologic information. (The above reagents were purchased from ServiceBio Company, Wuhan.)

A pathologist (M. H.) assessed the expression of PD-L1. PD-L1 positivity was defined as membrane staining in 20% of tumor cells.

PD-L1 positivity was defined as membrane-expressing PD-L1 tumor cells accounting for more than 5% of tumor cells [17]. PD-1 positivity was defined as membranous PD-1-expressing immunocytes, including lymphocytes and macrophages, accounting for more than 5% of all infiltrating lymphocytes. CD8 + TILs: Ten high-power fields (HPFs) were randomly selected for CD8 + TILs counting in each specimen, and the mean value of CD8 + TILs was taken as the CD8 + TIL infiltration degree of the specimen. The mean value of CD8 + TIL infiltration degree in all tumor specimens was taken as the cut-off point, and higher than the cut-off point was defined as high infiltration; otherwise, it was defined as low infiltration [18].

### Statistical analysis

All of the analyses were conducted using SPSS 22.0, and all of them were bilateral tests. In this study, 50 patients with HPV + OPSCC were selected and matched with 50 cases of HPV − OPSCC by PSM, including age, gender, smoking, tumor, primary site, treatment method, and clinical stage. All binary variables were analyzed by chi-square test (or Fisher’s test). Ordered categorical variables were analyzed by Mann–Whitney rank sum test. Kaplan–Meier method was used for univariate analysis of overall survival (OS) and disease-specific survival (DSS), and Cox proportional risk model was used for multivariate analysis. *P* < 0.05 was considered statistically different.

## Results

### Clinical and pathological parameters

In HPV positive group, there were 47 (94.00%) male and 3 (6.00%) female patients, of which 12 (24.00%) patients were younger than 55 years old. Thirty-one patients (62.00%) were smokers, and 28 (56.00%) patients were drinker. Forty-three patients (86.00%) with 0–1 point of Charlson comorbidity score and 7 (14.00%) patients were with higher. There were 16 (32.00%), 21 (42.00%), and 13 (26.00%) patients with high, moderate, and low differentiation of cancer in HPV positive group. Among these, 32 (64.00%) patients were with tonsil cancer and 18 (36.00%) patients were with cancer in root of tongue. There were 20 (40.00%) patients with stage I–II cancer and 30 (60.00%) patients with III–IV cancer. Of these, 28 (56.00%) patients received surgery and 22 (44.00%) did not. In HPV negative group, there were 45 (90.00%) male and 5 (10.00%) female patients, of which 13 (26.00%) patients were younger than 55 years old. Thirty-three patients (66.00%) were smokers, and 24 (48.00%) patients were drinker. Forty patients (80.00%) with 0–1 point of Charlson comorbidity score and 10 (20.00%) patients were with higher. There were 27 (54.00%), 13 (26.00%), and 10 (20.00%) patients with high, moderate, and low differentiation of cancer in HPV positive group. Among these, 33 (66.00%) patients were with tonsil cancer and 17 (34.00%) patients were with cancer in root of tongue. There were 14 (28.00%) patients with stage I–II cancer and 36 (72.00%) patients with III–IV cancer. Of these, 35 (70.00%) patients received surgery and 15 (30.00%) did not. There was no significant difference in these factors between the two groups (all *P* > 0.05; Table 1).

**Table 1:** Clinical and pathological characteristics

Variables	HPV positivity		HPV negativity		*P*
	(*n* = 50)		(*n* = 50)		
	*n*	%	*n*	%	
Gender					0.715
Male	47	94.00	45	90.00	
Female	3	6.00	5	10.00	
Age					0.817
≤ 55	12	24.00	13	26.00	
> 55	38	76.00	37	77.00	
Smoking					0.677
+	31	62.00	33	66.00	
−	19	38.00	17	34.00	
Alcohol					0.423
+	28	56.00	24	48.00	
−	22	44.00	26	52.00	
Comorbidity					0.424
0–1	43	86.00	40	80.00	
2–3	7	14.00	10	20.00	
Differentiation					0.060
High	16	32.00	27	54.00	
Moderate	21	42.00	13	26.00	
Low	13	26.00	10	20.00	
Site					0.834
Tonsil	32	64.00	33	66.00	
Root of tongue	18	36.00	17	34.00	
Stage					0.205
I–II	20	40.00	14	28.00	
III–IV	30	60.00	36	72.00	
Treatment					0.147
Surgery^a^	28	56.00	35	70.00	
Non-surgery^b^	22	44.00	15	30.00	

*HPV* human papillomavirus

^a^Surgery, surgery with chemotherapy or radiotherapy

^b^Non-surgery treatment, including radiotherapy and chemotherapy

### Immune phenotypic expression in OPSCC

**Fig. 1:**
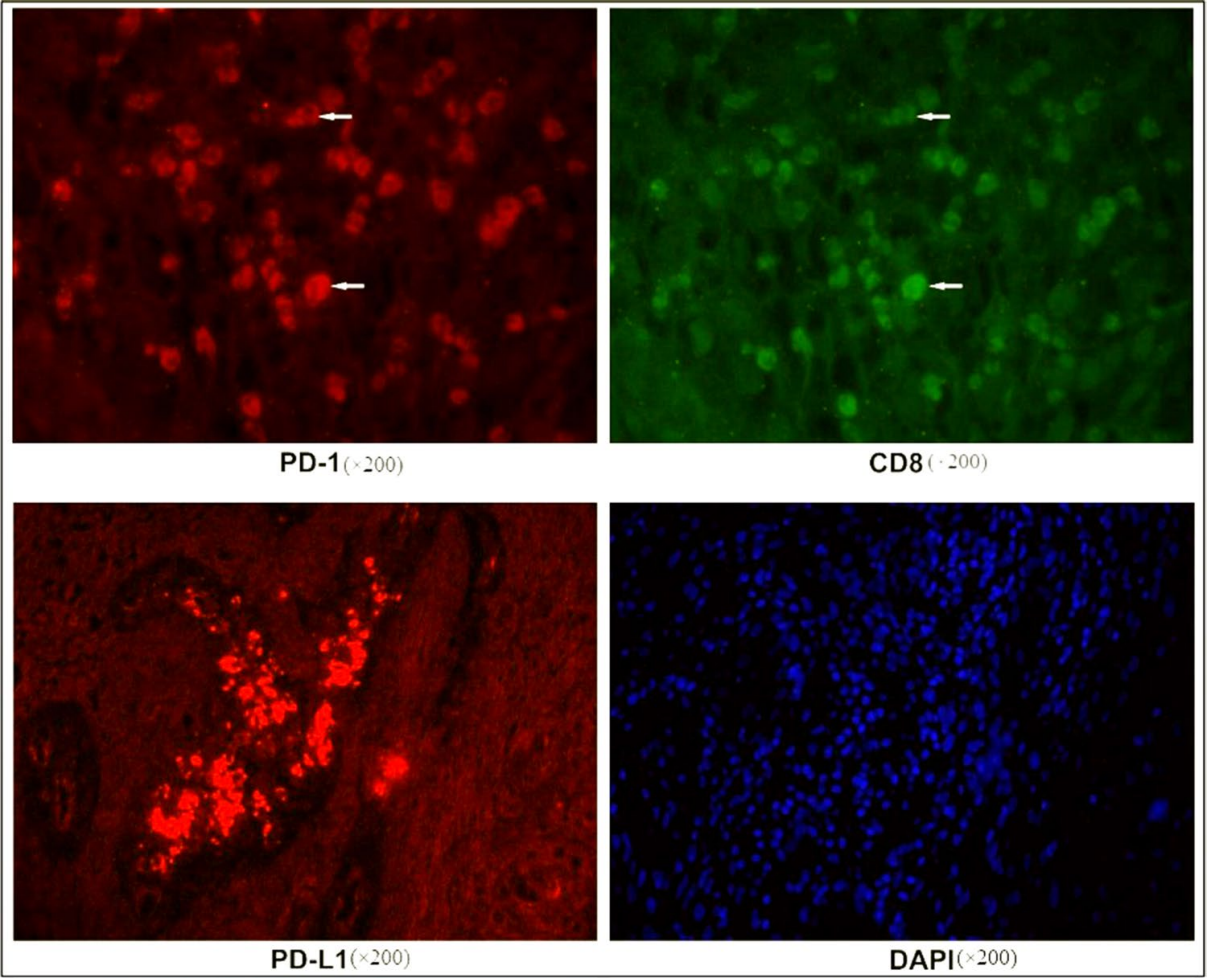
The immunofluorescence of CD8, PD-1, PD-L1. and DAPI. Arrows indicate the co-localization of PD-1 and CD8. PD-1, programmed death-1; PD-L1, programmed death ligand-1; magnification: × 200

**Fig. 2:**
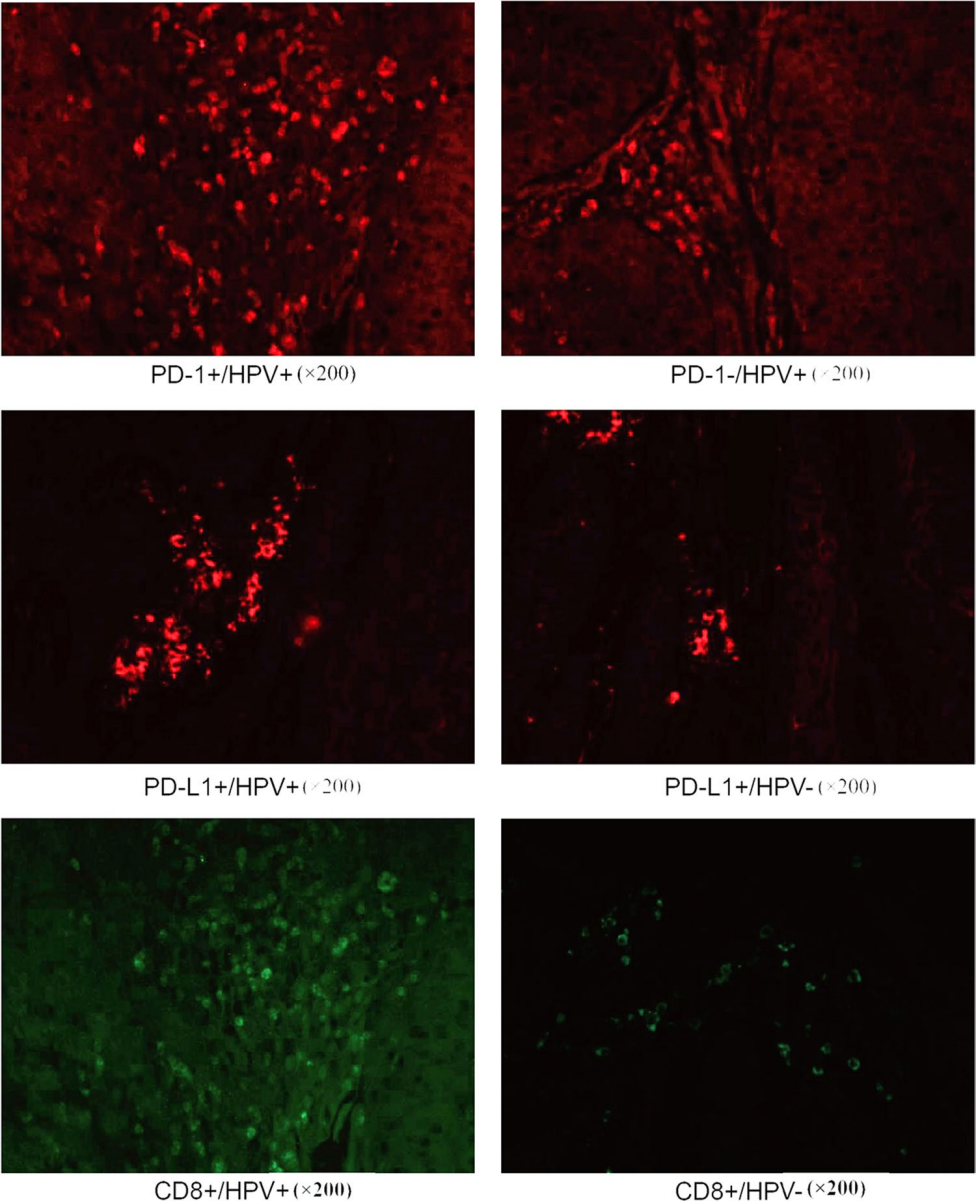
The immunofluorescence of PD-1, PD-L1, and CD8 in HPV + and HPV − samples. PD-1, programmed death-1; PD-L1, programmed death ligand-1; magnification: × 200

The expression rate of CD8 in HPV positive OPSCC was 72%; PD-1 positivity was 66%; PD-L1 was 70% (Fig. 1). The expressions of CD8, PD-1, and PD-L1 in HPV positive OPSCC were significantly higher than those of HPV negative patients (*P* = 0.039, *P* = 0.044, *P* = 0.005; Fig. 2). PD-L1 positivity in young patients was found higher than the elders (*P* = 0.020), and PD-1 was often seen in tonsillar squamous cell carcinomas (*P* = 0.052). CD8 + TILs were highly infiltrating in high differentiation (*P* = 0.029) and tonsillar squamous cell carcinomas (*P* = 0.004), but unrelated to other clinical pathological features including gender, smoking, alcohol, tumor stage, and complications (Table 2).

**Table 2:** The expression of TILs markers and HPV-related makers

Variables	CD8 + TILs High	CD8 + TILs Low	*P*	PD-1 +	PD-1 −	*P*	PD-L1 +	PD-L1 −	*P*
	(*n* = 62)	(*n* = 38)		(*n* = 56)	(*n* = 44)		(*n* = 56)	(*n* = 44)	
HPV			0.039			0.044			0.005
+	36	14		33	17		35	15	
−	26	24		23	27		21	29	
Gender			0.446			0.722			0.272
Male	58	34		52	40		53	39	
Female	4	4		4	4		3	5	
Age			0.234			0.163			0.020
≤ 55	13	12		11	14		9	16	
> 55	49	26		45	30		47	28	
Smoking			0.250			0.440			0.946
+	37	27		34	30		36	28	
−	25	11		22	14		20	16	
Alcohol			0.754			0.393			0.448
+	33	19		27	25		31	21	
−	29	19		29	19		25	23	
Comorbidity			0.423			0.415			0.797
0–1	50	33		48	35		46	37	
2–3	12	5		8	9		10	7	
Differentiation			0.029			0.451			0.234
High	31	12		22	21		22	21	
Moderate	22	12		22	12		23	11	
Low	9	14		12	11		11	12	
Site			0.004			0.052			0.128
Tonsil	47	18		41	24		40	25	
Root of tongue	15	20		15	20		16	19	
Stage			0.404			0.683			0.386
I–II	23	11		20	14		17	17	
III–IV	29	27		36	30		39	27	
Treatment			0.651			0.473			0.074
Surgery	38	25		37	26		31	32	
Non-surgery	24	13		19	18		25	12	

### Analyses of prognostic factors associated with survivals in OPSCC

The duration of follow-up ranged from 13 to 113 months, with a median of 67 and 47.5 months in the HPV + and HPV − groups, respectively. A total of 53 patients died during the follow-up period, of whom 45 patients died from OPSCC and 8 patients died of other complications such as myocardial infarction, ruptured aneurysm, and primary lung cancer and hepatoma. The 5-year OS rate was 66% and 40% in the HPV + and HPV − groups, respectively (*P* = 0.003), and the 5-year DSS rate was 73% and 44% in these two groups, respectively (*P* = 0.001). The Cox multivariate regression analysis showed that HPV positivity (HR = 0.46, 95% CI: 0.25–0.85, *P* = 0.013), high infiltration of CD8 + TILs (HR = 0.40, 95% CI: 0.21–0.75, *P* = 0.004), PD-L1 positivity (HR = 0.41, 95% CI: 0.21–0.79, *P* = 0.007), smoking (HR = 2.04, 95% CI: 1.04–3.98, *P* = 0.037), complications (HR = 2.39, 95% CI: 1.22–4.71, *P* = 0.012), the low degree of differentiation (HR = 2.83, 95% CI: 1.34–5.97, *P* = 0.006), and treatment method (HR = 0.44, 95% CI: 0.21–0.92, *P* = 0.029) were independent prognostic factors of DSS. The multivariate analysis of OS was similar to the DSS. Positive HPV, high infiltration of CD8 + TILs, PD-L1, complications, and degree of differentiation were all independent prognostic factors, but smoking and treatment regimen were no longer independent factors. Besides, the expression of PD-1 had no effect on prognosis (Table 3).

**Table 3:** Prognostic factors related to OSCC

	Univariable analysis (P)	Multivariable analysis		
		HR	95% CI	*P*
OS				
HPV (+ vs. -)	0.002	0.459	0.249–0.847	0.013
CD8 + TILs (High vs. Low)	< 0.001	0.395	0.209–0.747	0.004
PD-1 (+ vs. −)	0.118	1.820	0.962–3.440	0.065
PD-L1 (+ vs. −)	< 0.001	0.410	0.214–0.786	0.007
Gender (male vs. female)	0.121	-	-	-
Age (≤ 55 vs. > 55)	0.234	-	-	-
Smoking (+ vs. −)	0.002	1.888	0.969–3.680	0.062
Alcohol (+ vs. −)	0.267			
Complication (+ vs. −)	0.017	2.393	1.216–4.71	0.012
Differentiation (high vs. medium)	0.019	1.275	0.598–2.719	0.530
Differentiation (high vs. low)		2.829	1.340–5.972	0.006
Site (tonsil vs. root of tongue)	0.641	-	-	-
Treatment (surgery vs. non-surgery)	0.113	-	-	-
Stage (I–II vs. III–IV)	0.170	-	-	-
DSS				
HPV (+ vs. −)	0.002	0.430	0.218–0.846	0.014
CD8 + TILs (high vs. low)	< 0.001	0.349	0.173–0.702	0.003
PD-1 (+ vs. −)	0.071	1.359	0.612–3.022	0.451
PD-L1 (+ vs. −)	< 0.001	0.409	0.194–0.864	0.019
Gender (male vs. female)	0.145	-	-	-
Age (≤ 55 vs. > 55)	0.612	-	-	-
Smoking (+ vs. −)	0.057	2.036	1.042–3.976	0.037
Alcohol (+ vs. −)	0.601			
Complication (+ vs. −)	0.164	2.491	1.113–5.578	0.026
Differentiation (high vs. medium vs. low)	0.021	1.523	0.652–3.559	0.331
		3.196	1.441–7.087	0.004
Site (tonsil vs. root of tongue)	0.666	-	-	-
Treatment (surgery vs. non-surgery)	0.056	0.444	0.214–0.921	0.029
Stage (I–II vs. III–IV)	0.074	-	-	-

*OSCC* oropharyngeal squamous cell carcinoma, *HPV* human papillomavirus, *CI* confidence interval, *HR* hazard ratio, *OS* overall survival, *DSS* disease-specific survival rate, *TILs* tumor infiltrating lymphocytes, *PD-1* programmed death-1, *PD-L1* programmed death ligand-1

### Association of immune indicators with survivals

**Fig. 3:**
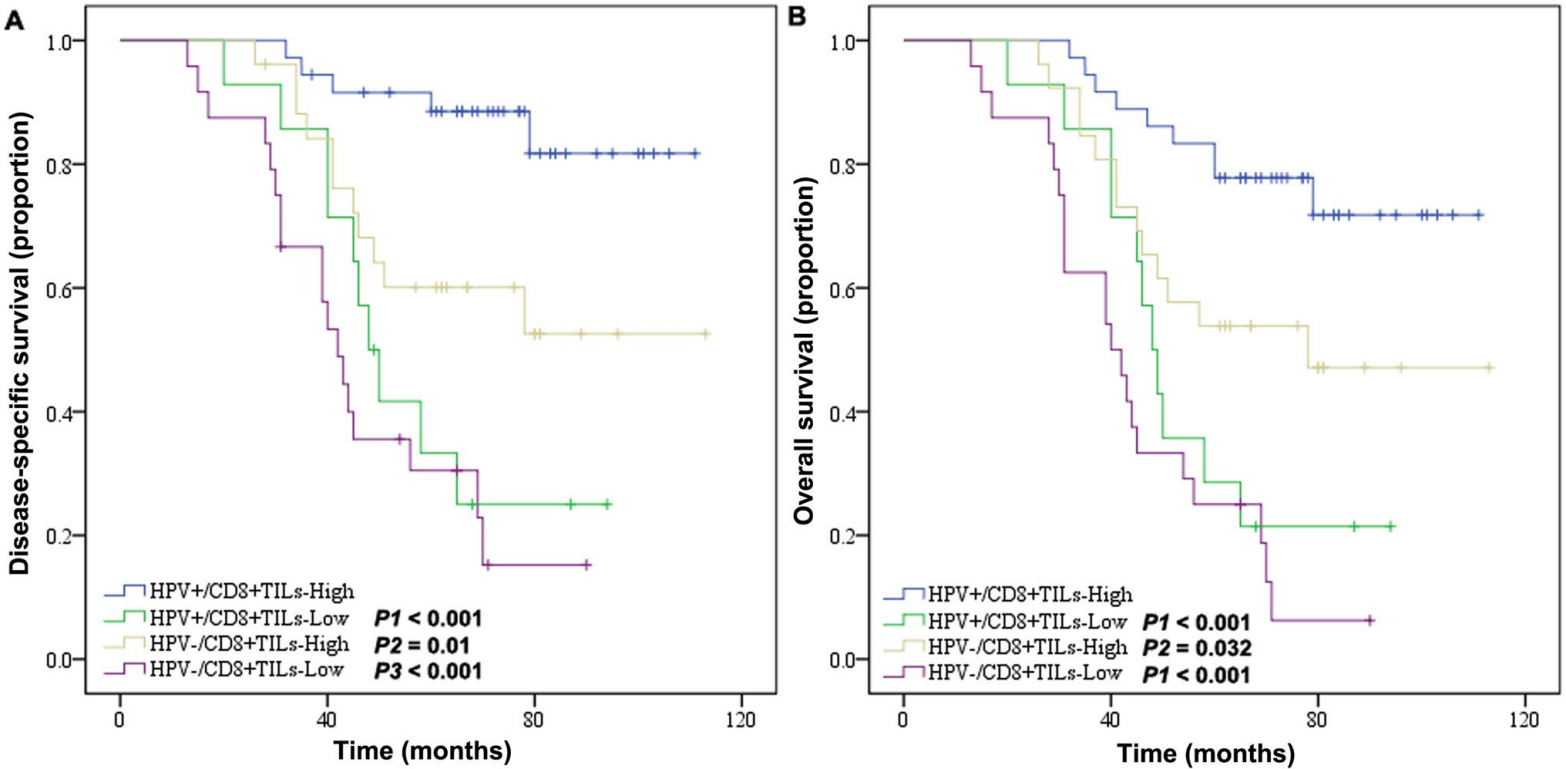
Kaplan–Meier curve of HPV infection status and CD8 + TIL infiltration in patients with OPSCC: **A** Disease-specific survival rate. **B** Overall survival. OPSCC, oropharyngeal squamous cell carcinoma; TILs, tumor infiltrating lymphocytes

**Fig. 4:**
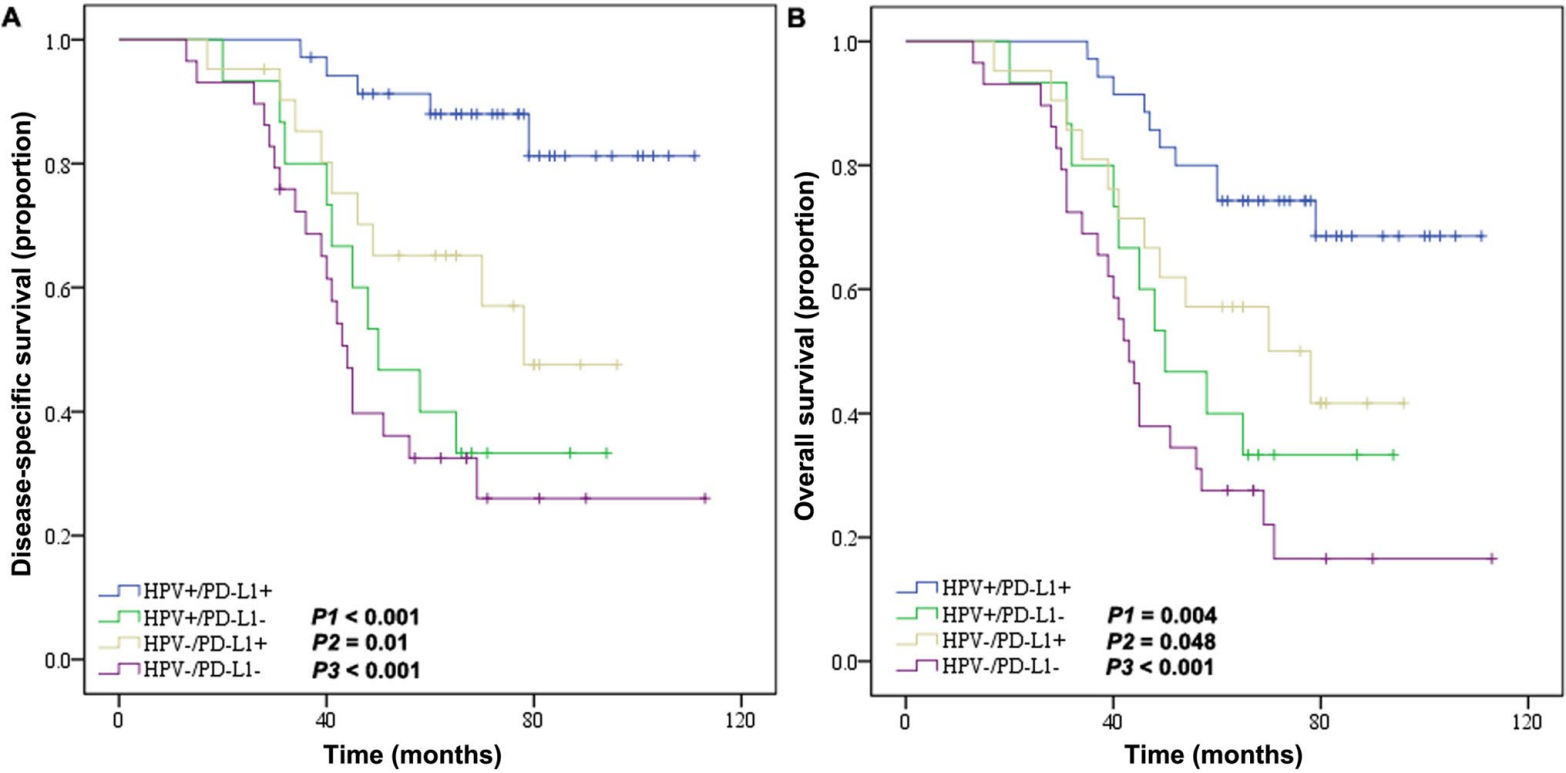
Kaplan–Meier curve of HPV infection status and PD-L1 expression on the prognosis of patients with OPSCC: **A** Disease-specific survival rate. **B** Overall survival. HPV, human papillomavirus; PD-1, programmed death-1; OPSCC, oropharyngeal squamous cell carcinoma

**Fig. 5:**
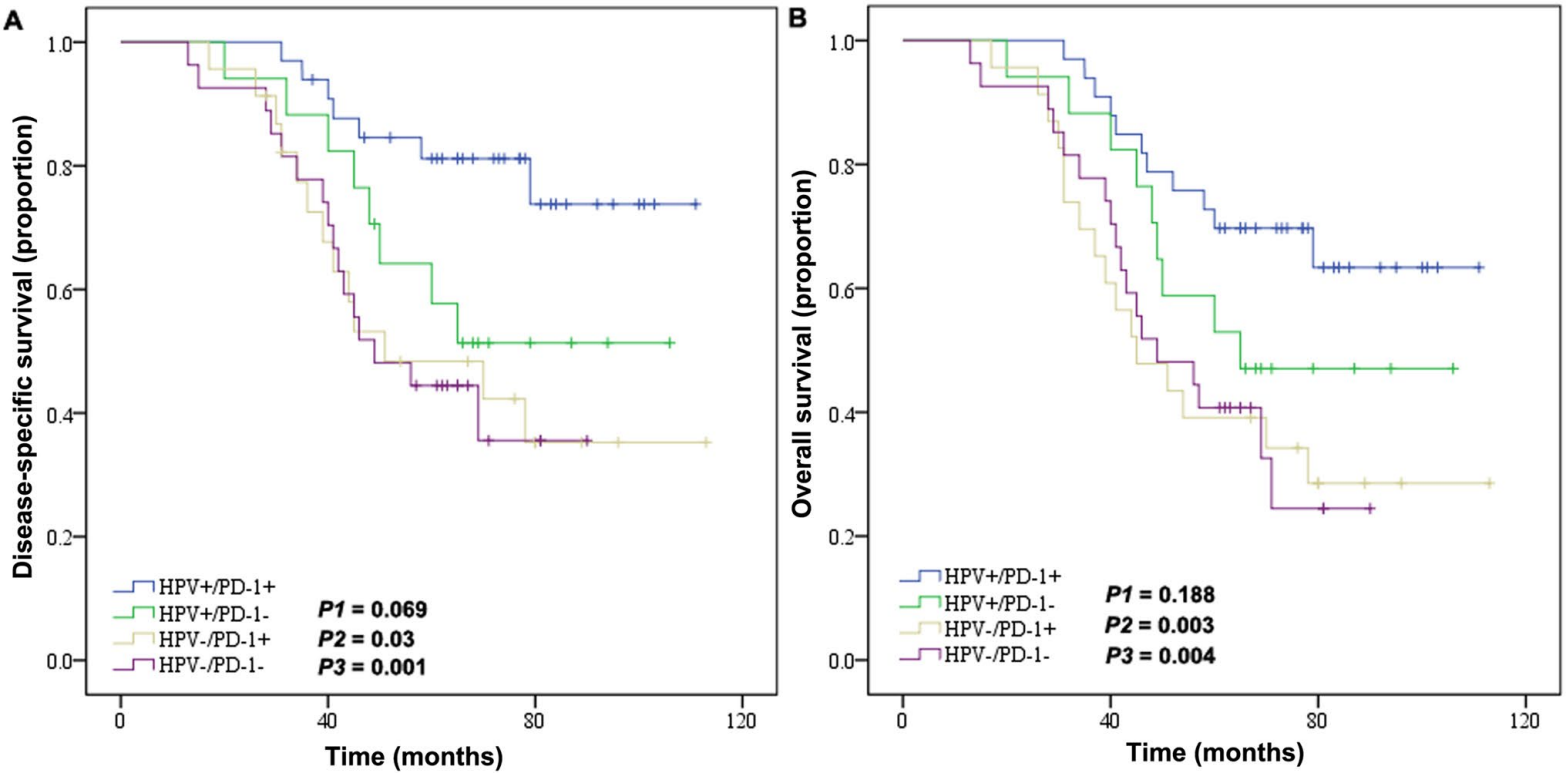
Kaplan–Meier curve of HPV infection status and PD-L1 expression on the prognosis of patients with OPSCC: **A** Disease-specific survival rate. **B** Overall survival. HPV, human papillomavirus; PD-L1, programmed death ligand-1; OPSCC, oropharyngeal squamous cell carcinoma

In order to further analyze the prognostic differences of various immune indicators under different HPV infection status, we conducted a stratified analysis of the two groups of patients. The results showed that patients with high HPV + /CD8 + TIL infiltration were more likely to have a favorable prognosis than those with low HPV + /CD8 + TILs (DSS, *P* < 0.001; OS, *P* < 0.001), high expression of HPV − /CD8 + TILs (DSS, *P* = 0.010; OS, *P* = 0.032), and low expression of HPV − /CD8 + TILs (DSS, *P* < 0.001; OS, *P* < 0.001) (Fig. 3). Similarly, patients with HPV + /PD-L1 + were more inclined to have a better prognosis than those with HPV + /PD-L1 − (DSS, *P* < 0.001; OS, *P* = 0.004), HPV − /PD-L1 + (DSS, *P* = 0.010; OS, *P* = 0.048) and HPV − /PD-L1 − (DSS, *P* < 0.001; OS, *P* < 0.001) (Fig. 4). For the stratified analysis of HPV and PD-1, HPV + /PD-1 + patients had better outcome only than HPV − /PD-1 + (DSS, *P* = 0.03; OS, *P* = 0.003) and HPV − /PD-1 − (DSS, *P* = 0.001; OS, *P* = 0.004) (Fig. 5).

## Discussion

HPV infection is one of the important pathogenic factors of oropharyngeal cancer, causing about 31% of oropharyngeal cancer worldwide [19]. HPV + oropharyngeal cancers are usually more advanced and poorly differentiated than HPV − oropharyngeal cancers [20, 21]. Since then, more and more studies have suggested that the prognosis of patients with HPV + oropharyngeal carcinoma is significantly improved after standard anti-HPV therapy. In this study, 50 pairs of patients with HPV-positive and HPV-negative oropharyngeal cancer were enrolled by PSM. The results further confirmed that HPV-positivity is an independent factor for good prognosis of OPSCC in the Chinese population.

However, the mechanism about that patient with HPV + OPSCC has a better prognosis remains unclear. Some scholars attributed the better prognosis to increased sensitivity to chemoradiotherapy [22]. Patients with HPV + tumors who underwent surgery alone can still have an improved prognosis compared to patients with comparable HPV − tumors [23]. Williams et al. found that the proliferation rate of HPV + tumor cells expressing E6/E7 protein was significantly lower than that of HPV − tumor cells in immunoactive mice, but the same experimental results were not found in immunodeficient nude mice. Therefore, it was speculated that the better clinical prognosis of HPV + tumor may be related to the adaptive immunity of the organism [24]. Immunotherapy, especially immune checkpoint inhibitors targeting PD-1/PD-L1, has shown promising results in a variety of tumors (such as lung cancer [25], melanoma [26], kidney cancer [27], bladder cancer [28], colon cancer [29]), and has also achieved good efficacy in OPSCC [30]. It is important to note that only a small proportion of patients show lasting efficacy. Checkmate 141 and other clinical studies found that patients with head and neck cancer positive for PD-L1 had better efficacy after receiving the PD-1 inhibitor nivolumab [31]. Therefore, it is necessary to detect the expression levels of PD-1, PD-L1, and CD8 + TILs in the Chinese population of patients with OPSCC, so as to provide a theoretical basis for better clinical prognosis of HPV + patients and provide baseline data for the application of PD-1/PD-L1 in head and neck tumors.

Currently, there are a number of studies on the relationship between HPV-infection-related tumors and their immunophenotypes. One study showed that patients with HPV + cervical cancer had high serum levels of PD-L1 which could be diagnostic indicator for identifying HPV + cervical cancer [32]. Another study demonstrated that HPV infection and p16 overexpression were significantly associated with PD-LI expression in epithelial ovarian cancer, through the cooperative roles of dendritic cells (DCs) and IFN-γ [33]. However, Wessely found that HPV infection status and PD-L1 expression were not correlated, PD-L1 expression status was an independent prognostic marker for survival of patients with anal squamous cell carcinoma (*P* = 0.012) [34], and a meta-analysis demonstrated that in anogenital SCC, PD-L1 positivity had to do with a worse outcome, which might attribute to advanced age, higher tumor grade, lymph node metastasis, and HPV negativity, while in oropharynx cancer, PD-L1 expression was related to better prognosis for the reason that PD-L1 was less frequent in the aged and negative HPV status [35]. Balermpas et al. conducted a research of 161 patients with advanced OPSCC from multi-centers and found that the expression of PD-1 and PD-L1 in HPV + tumors was higher than that in HPV- tumors. High infiltration of CD8 + TILs and positive expression of PD-L1 were independent prognostic indicators for patients with OPSCC [36]. After detecting various related receptors on the surface of TILs, Kansy et al. found that the expression of PD-1 on the surface of CD8 + TILs (PD-1 + /CD8 + TILs) in HPV + OPSCC was significantly different from that in HPV − tumor tissues, while there was no significant difference in the expression of PD-1 on other types of TILs (such as CD3 + TILs, CD4 + TILs and CTLA-4 + TILs). Moreover, patients with high infiltration of PD-1 + /CD8 + TILs had worse disease-free survival and higher risk of recurrence [37, 38]. Another study, involving 133 cases of OPSCC, showed no significant difference in PD-L1 expression between HPV + and HPV − tumors, and the presence or absence of PD-L1 expression did not affect the prognosis of patients [37]. Solomon and his team found that in HPV + OPSCC, the presence of intra-tumoral PD-L1 immune cells or CD8 + TILs is of prognostic value [13]. Later, they confirmed the role of immune biomarkers such as PD-L1 to identify subgroups of HPV + OPSCC patients with an excellent outcome that may be suitable for trials evaluating de-intensification of therapy [39]. Moreover, another research found that HPV-positivity correlated with increased immune cytolytic activity and a T cell-inflamed gene expression profile, which provided evidence that HPV status can be used to predict the effectiveness of PD-1 inhibitors in OPSCC, independently of PD-L1 expression and TMB, and probably results from an inflamed immune microenvironment induced by HPV infection [40]. In this study, it was found that the expression of PD-L1 in OPSCC was significantly correlated with HPV 16 infection status. However, Jeong found that high expression of PD-L1 might suggest a poorer outcome for OPSCC patients, especially in the HPV 16 positive subgroup [41]. This study concluded that high expression of PD-L1 was related to HPV positivity. It was obvious that patients with HPV + OPSCC had better prognosis. Therefore, the good prognostic value of PD-L1 mainly depends on its correlation with HPV positivity.

These contradictory results may be partly due to the small sample size and partly due to the lack of uniformity of the analytical methods, including variability in the immunohistological tests between observers, the lack of standardized antibodies to determine PD-L1 expression, and the different thresholds for defining positive expression. At the same time, the expression of PD-L1 in tumor cells may change dynamically in different stages of the disease, and the detection results may be affected by the time of biopsy. Moreover, tumors are heterogeneous, and the expression of PD-L1 may be different in different locations of the same lesion and among different lesions. The results of this study suggested that positive PD-L1 was an independent prognostic factor for improving OS and DSS. Meanwhile, patients with HPV + /PD-L1 + were more likely to have a significantly better prognosis than those with HPV + /PD-L1 − , HPV − /PD-L1 + , and HPV − /PD-L1 − .

These contradictions and inconsistencies may be related to the following reasons: (1). Virus-associated tumors are caused by virus-associated oncoproteins, and usually, the tumor mutation load is low or moderate. The emergence of corresponding neoantigens to virus infection may cause stronger immune response. Many virus-associated tumors show strong immune response and overexpression of PD-L1 [42]; (2). After the immune system is activated by tumor specific antigens and tumor-associated antigens, the expression of PD-L1 in tumor cells is upregulated. The constitutive expression of PD-L1 may represent a previous endogenous anti-tumor immune response, which is slowed but failed to prevent tumor growth completely, and can be rejuvenated by PD-1/PD-L1 inhibitors [43]; (3). Studies have suggested different mechanisms of PD-L1 overexpression, including dynamic IFNγ [44] or oncogene activation [45]. In the former case, PD-L1 overexpression caused by elevated IFNγ is part of acquired immunity and is usually associated with immune cell infiltration, showing localized overexpression [46]. Oncogene-activation-associated PD-L1 overexpression is often diffuse due to the lack of lymphocyte infiltration [47, 48]. The overexpression of PD-L1 caused by different mechanisms may be related to the different efficacy of immune checkpoint inhibitors.

### Limitations

Our study had several limitations. First, immunofluorescence staining is a semi-quantitative detection, which cannot perfectly display the expression level of each marker. Second, even though a relatively large cohort was analyzed, compared to previous studies, because of the favorable prognosis of OPSCC, only a few events occurred in the entire cohort. This might limit the power of the study to determine the prognostic role of PD-L1 expression. Third, the inherent nature of a retrospective study makes it impossible to explore any dynamic changes in PD-L1 expression.

## Conclusion

In conclusion, the results of this study confirmed that the prognosis of patients with HPV + OPSCC is significantly better compared with those with HPV − . CD8 + TILs, PD-L1, and PD-1 were more highly expressed in patients with HPV + OPSCC. The infiltration degrees of CD8 + TILs and PD-L1 expression were independent factors for the prognosis. The favorable prognosis of patients with HPV + OPSCC might be related to different immune microenvironment. This study also provided a theoretical basis and baseline data for the application of immune checkpoint inhibitors in head and neck tumors.

## Data Availability

The datasets in this study are available from the corresponding author on reasonable request.
